# Vitamin D Status in Patients with Primary Antiphospholipid Syndrome (PAPS): A Systematic Review and Meta-Analysis

**DOI:** 10.3390/antib13010022

**Published:** 2024-03-13

**Authors:** Md Asiful Islam, Saleh Ahmed, Shabiha Sultana, Sayeda Sadia Alam, Tareq Hossan, Wesam Gouda, Faisal Alsaqabi, Rosline Hassan, Przemysław J. Kotyla

**Affiliations:** 1 WHO Collaborating Centre for Global Women’s Health, Institute of Metabolism and Systems Research, College of Medical and Dental Sciences, University of Birmingham, Birmingham B15 2TT, UK; 2 Center for Biotechnology and Genomic Medicine, Medical College of Georgia, Augusta University, Augusta, GA 30912, USA; 3 Department of Cellular Biology and Anatomy, Medical College of Georgia, Augusta University, Augusta, GA 30912, USA; 4 Department of Biochemistry & Molecular Biology, Jahangirnagar University, Savar, Dhaka 1342, Bangladesh; 5 Department of Internal Medicine, Division of Oncology, Washington University School of Medicine in St. Louis, St. Louis, MO 63110, USA; 6 Department of Rheumatology, Al-Azhar University Hospital, Assiut 71524, Egypt; drwesamgouda@yahoo.com; 7 Department of Rheumatology, Al-Sabah Hospital, Kuwait City P.O. Box 4078, Kuwait; 8 Department of Haematology, School of Medical Sciences, Universiti Sains Malaysia, Kota Bharu 16150, Kelantan, Malaysia; roslin@usm.my; 9 Department of Internal Medicine, Rheumatology and Clinical Immunology, Faculty in Katowice, Medical University of Silesia, 40-635 Katowice, Poland

**Keywords:** vitamin D, APS, primary antiphospholipid syndrome, systematic review, meta-analysis

## Abstract

Primary antiphospholipid syndrome (PAPS) is a systemic autoimmune disorder, characterised by consistently high levels of antiphospholipid antibodies, thrombosis, and/or pregnancy morbidity. Due to various suspected causes, deficient or insufficient levels of vitamin D in the serum have been reported in patients with PAPS; however, the reports have been sporadic and inconclusive. This systematic review and meta-analysis aimed to comprehensively evaluate the serum vitamin D levels in patients with PAPS compared to controls. A protocol was registered in PROSPERO (Registration No. CRD42019132128) and a systematic literature search was conducted through Google Scholar, PubMed, Web of Science, Scopus, and ScienceDirect databases without restricting language and year. Pooled prevalence, mean difference (MD), and odds ratio (OR) along with 95% confidence intervals (CI) were determined by using a random effects model. Study quality was assessed by the Joana Brigg’s Institute (JBI) protocol and publication bias was evaluated by a trim and fill funnel plot, Begg’s, and Egger’s tests. The pooled prevalence of vitamin D deficiency and insufficiency was found to be 32.2% [95% CI: 16.3–48.2] and 61.5% [95% CI: 40.2–82.8], respectively. Serum levels of vitamin D were considerably lower in the PAPS patients compared to controls (MD: −5.75, 95% CI: −9.73 to −1.77; *p* = 0.005). Multiple sensitivity analyses showed that the results remained statistically significant, demonstrating the robustness of this meta-analysis. No significant publication bias was detected in determining the MD of serum vitamin D levels in PAPS and controls. In conclusion, PAPS patients had greater rates of vitamin D deficiency or insufficiency, higher frequency of thrombosis, and lower serum vitamin D levels than healthy individuals.

## 1. Introduction

A systemic autoimmune disorder called antiphospholipid syndrome (APS) is characterised by the occurrence of arterial, venous, or microvascular blood clotting, complications during pregnancy, or non-thrombotic symptoms in individuals who have persistent antiphospholipid antibodies (aPLs) [1]. According to the new 2023 ACR/EULAR APS classification criteria, it needs a positive antiphospholipid antibody (aPL) test within 3 years of identifying an aPL-associated clinical criterion as the initial requirement. This is followed by additional weighted criteria with a scoring range of 1 to 7 points each. The subjects are categorised into six clinical areas (macrovascular venous thromboembolism, macrovascular arterial thrombosis, microvascular, obstetric, cardiac valve, and hematologic) and two laboratory areas (lupus anticoagulant functional coagulation assays, and solid-phase enzyme-linked immunosorbent assays for IgG/IgM anticardiolipin and/or IgG/IgM anti–β2-glycoprotein I antibodies). Patients who score at least 3 points in both the clinical and laboratory areas are categorised as having APS [1]. APS is considered to be an idiopathic disease, and the exact cause or mechanism of the disease is yet to be discovered. Nevertheless, APS has been established to be a polygenic disorder where immunogenic and genetic factors contribute to the pathogenesis of the disease [2,3].

Vitamin D is a crucial steroid hormone that has been extensively studied for its impact on skeletal health, cardiovascular function, and mineral metabolism. In recent years, there has been a growing recognition of the ability of vitamin D to exert non-classical actions, such as immunomodulatory effects [4,5,6]. The mere existence of aPLs does not automatically make a patient more likely to experience thrombotic events and the clinical antiphospholipid syndrome (APS). To elucidate this phenomenon, a theoretical framework known as the “two-hit” hypothesis has been adopted to account for thrombosis [7]. The initial “first hit” refers to the existence of aPLs that are recognised for their ability to facilitate thrombosis via various mechanisms and the deficiency of vitamin D may potentially serve as an additional “second hit” for thrombotic events, supported by reports in multiple studies [8,9,10,11]. The deficiency of vitamin D initiates the activation of two crucial components involved in blood coagulation, namely tissue factor (TF) and plasminogen activator inhibitor-1 (PAI-1) [9,11,12]. In addition, vitamin D deficiency has been shown to decrease the formation of thrombomodulin, a glycoprotein with anticoagulant properties [10]. Consequently, this phenomenon leads to an elevation in platelet aggregation and the activation of pro-inflammatory cytokines such as NF kappa beta, TGF-beta, and TNF-alpha, ultimately leading to the development of inflammation and thrombosis [8,13]. The determination of vitamin D levels in the bloodstream is mostly done through the estimation of serum levels of 25-hydroxyvitamin D (25(OH)D), as vitamin D is the predominant circulating vitamin with a half-life of about three weeks. There is no agreement on the ideal, insufficient, or deficient levels of 25(OH)D; nevertheless, a serum level of >30 ng/mL is considered adequate in most of the studies [14,15]. A significant global occurrence of low serum vitamin D levels has been documented, affecting both healthy individuals and individuals diagnosed with various medical conditions like rheumatic diseases [16]. Additionally, thrombotic events were found to be increased with decreased levels of serum vitamin D in patients diagnosed with antiphospholipid syndrome (APS) [11]. In light of the growing body of evidence suggesting a correlation between vitamin D deficiency and vein thrombosis in antiphospholipid syndrome (APS), the 16th International Congress on Antiphospholipid Syndrome endorses the use of vitamin D supplementation as an additional therapeutic approach in the management of APS patients who also exhibit vitamin D deficiency [7]. 

Although there is an increasing amount of research suggesting an association between low serum levels of vitamin D and the clinical manifestations of APS in individuals [11,17,18,19,20], it is random and inconclusive. Therefore, we conducted this systematic review and meta-analysis to comprehensively assess the serum vitamin D levels in PAPS patients in comparison to healthy individuals.

## 2. Materials and Methods

### 2.1. Guidelines and Protocol

This systematic review and meta-analysis (SRMA) followed the guidelines outlined by Preferred Reporting Items for Systematic Review and Meta-Analysis (PRISMA) [21] and Meta-analysis of Observational Studies in Epidemiology (MOOSE) [22] (Tables S1 and S2). The study protocol was registered in the PROSPERO database under the reference number CRD42019132128.

### 2.2. Eligibility Criteria and Search Strategies

Observational studies such as cross-sectional, case-control, or cohort were included where levels of vitamin D in the serum were assessed in adult patients with PAPS. Non-human studies, review articles, case reports, editorials, and comments were excluded from this SRMA. To identify relevant studies, a comprehensive literature search was conducted utilizing relevant keywords across five electronic databases, namely PubMed, Google Scholar, Web of Science, Scopus, and ScienceDirect until 31 May 2023. No restriction on language and year of publication was imposed. Moreover, a systematic search was performed utilizing citations from retrieved papers to identify any further appropriate studies that may have been overlooked. The comprehensive search methodology for all databases is outlined in Table S3.

### 2.3. Study Screening and Selection

The references of the relevant studies were initially saved and managed by EndNote X8. Following the elimination of duplicate studies, a group of four authors (M.A.I., S.A., S.S., and S.S.A.) independently identified the appropriate studies. Based on the eligibility criteria, the titles and abstracts were first screened to identify the potential studies and then subjected to a full-text review. Any disagreements or uncertainty were resolved by discussion with other researchers. The inclusion of research data or findings in multiple articles by the same author, although in different formats or names, was considered only once. 

### 2.4. Quality Assessment

Two independent authors (M.A.I. and S.S.A.) assessed the methodological quality of each study using the Joana Brigg’s Institute (JBI) checklist for cross-sectional and case-control studies. The evaluation was conducted using four response choices: ‘Yes’, ‘No’, ‘Unclear’, and ‘Not applicable’. The ultimate score of each article was determined based on the percentage of ‘Yes’ responses. Studies were classified as high risk of bias (low quality), moderate risk of bias (moderate quality), or low risk of bias (high quality) based on the overall scores of ≤49%, 50–69%, and ≥70%, respectively [23,24]. 

### 2.5. Data Extraction

The pertinent data from each eligible study was recorded in a predetermined Excel file by four authors (M.A.I., S.A., S.S., and S.S.A.). The following information and data were extracted from the included studies: country and latitude, age of the participants, study population, disease duration, APS diagnostic criteria, measurement techniques, vitamin D deficiency and insufficiency (%), cut-off values for vitamin D deficiency and insufficiency levels (ng/mL), as well as summary outcomes. To resolve discrepancies, all the authors were involved in the discussion and, if unresolved, the corresponding authors were contacted specially in the case of missing or unclear data.

### 2.6. Data Analyses

Forest plots were generated representing the pooled prevalence, odds ratio (OR), and mean difference (MD) of vitamin D deficiency and insufficiency with a corresponding 95% confidence interval (CI) using the random effects model. The unit of measurement for serum vitamin D level was recorded as nanograms per millilitre (ng/mL). In cases where a study presented the amount of vitamin D in serum as nmol/L, the values were adjusted to ng/mL using the conversion factor of 1 nmol/L = 0.4 ng/mL. The heterogeneity among the studies was evaluated by *I*^2^ statistics where *I*^2^ values >75%, 50–70%, and <50% were indicative of considerable, moderate, and low heterogeneity, respectively. The Cochran’s Q-test was utilised to determine the significance of heterogeneity [25]. However, the heterogeneity analysis regarding a small number of studies (usually <10 studies) is discouraged. This is due to the fact that even minimal levels of interstudy variance would yield a substantial level of variation and biases [26]; thus, in the context of this SRMA, the utilisation of *I*^2^ or *χ*^2^ tests for assessing heterogeneity would be inappropriate. The presence of publication bias was assessed by both visual examination of a funnel plot and objective analysis using Egger’s and Begg’s regression tests (*p* < 0.10). Subgroup analysis was performed on the prevalence of thrombotic events in vitamin D deficient PAPS. In addition, the robustness of the combined estimate was evaluated by conducting sensitivity analysis employing the following approaches: (i) exclusion of studies with small sample sizes (*n* < 100); (ii) exclusion of studies of low and moderate quality (those with high and moderate risk of bias); and (iii) utilisation of a fixed-effects model. The analyses and graphs were produced using the metafor (version 3.8-1) packages of R (version 4.2.2) and metaprop codes in the meta (version 6.1-0) within the RStudio (version 1.2.5033) environment [27]. Additionally, RevMan (version 5.4) [28] was also utilised for this purpose.

## 3. Results

### 3.1. Study Selection

**Figure 1:**
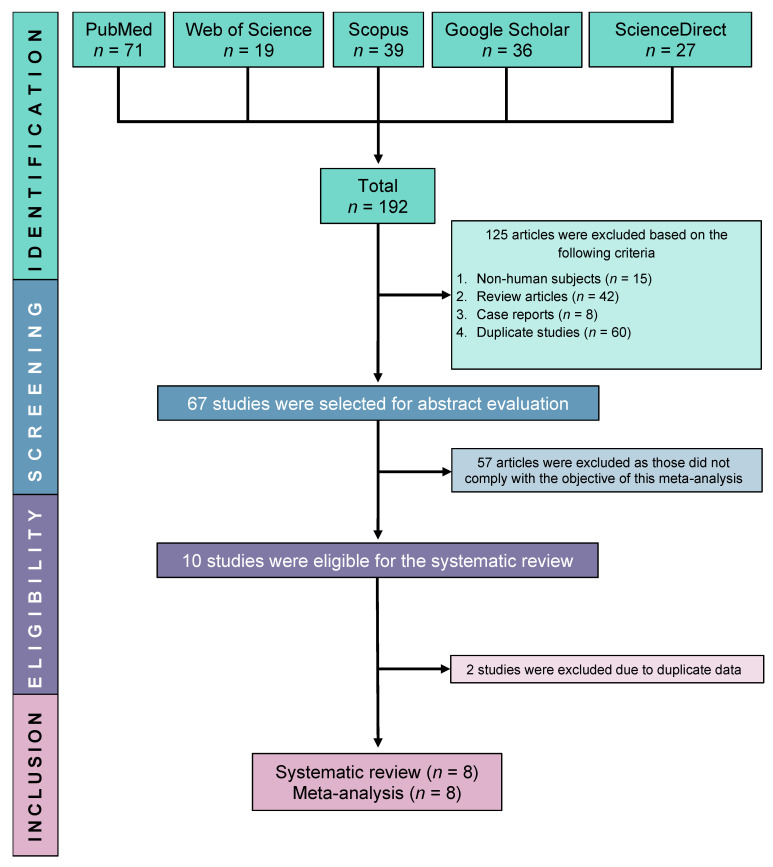
PRISMA flow diagram of study selection.

Initially, we were able to find a total of 192 relevant studies by searching in five electronic databases: ScienceDirect, PubMed, Google Scholar, Scopus, and Web of Science. Following the exclusion of 125 ineligible studies, 67 studies were further screened for full-text eligibility. Finally, eight studies were fully eligible and included in this SRMA (Figure 1). 

### 3.2. Characteristics of Included Studies

Among the eight included studies, five were case-control [11,17,20,29,30] and three were cross-sectional designed studies [31,32,33] reporting serum levels of vitamin D in a total of 1195 participants (PAPS patients = 637 and Healthy controls = 558). The participants of six studies were from seven European countries such as Italy, Serbia, Belarus, Israel, Germany, Hungary, and Spain [11,29,30,31,32,33], and two studies were from Brazil [20,32]. The concentration of vitamin D in the serum was estimated using a chemiluminescent immunoassay [11,20,29,30,31], radio immunoassay [17,32], and ELISA [33]. Various threshold values were used to define vitamin D deficiency (i.e., 10, 15, or 20 ng/mL) and insufficiency (i.e., 10, 15, 20–30 ng/mL). Five [11,29,30,31,33] and three [17,20,32] of the studies followed the 2006 Sydney and 1999 Sapporo APS classification criteria, respectively. The major features of the included studies are summarised in Table 1.

### 3.3. Meta-Analyses

**Figure 2:**
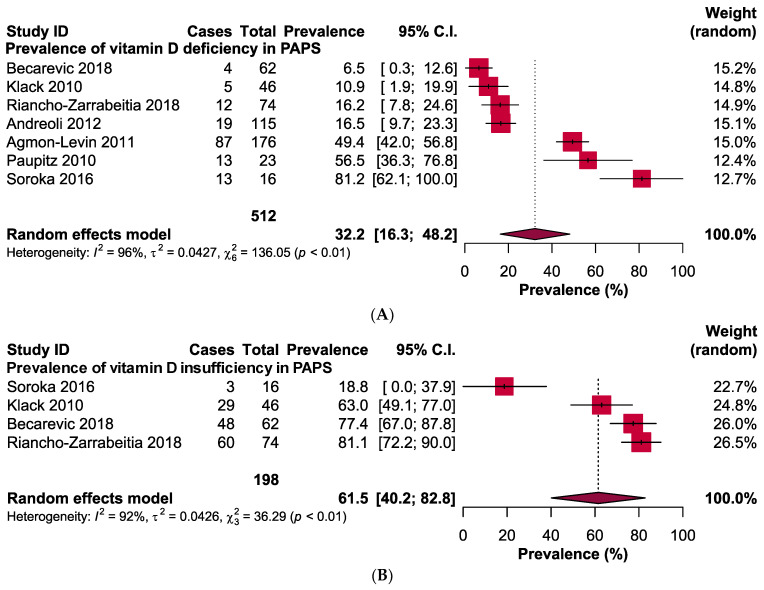
Prevalence of vitamin D (**A**) deficiency and (**B**) insufficiency in patients with primary antiphospholipid syndrome.

**Figure 3:**
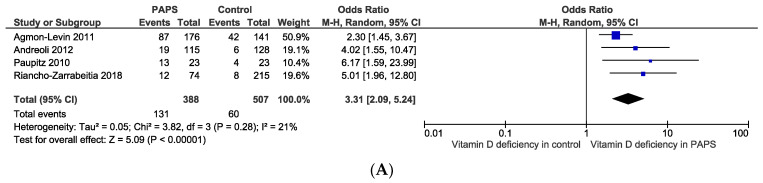

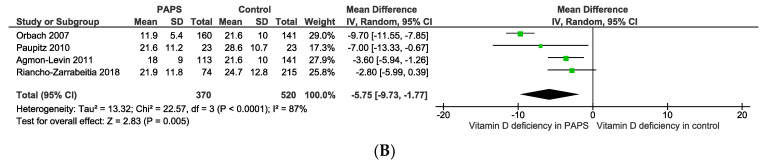
(**A**) Odds ratio and (**B**) mean difference of vitamin D deficiency in patients with primary antiphospholipid syndrome compared to healthy controls.

The pooled prevalence of vitamin D deficiency and insufficiency was 32.2% [95% CI: 16.3–48.2] and 61.5% [95% CI: 40.2–82.8] in patients with PAPS, respectively (Figure 2). The odds ratio of vitamin D deficiency in PAPS was significantly (*p* < 0.00001) higher (OR: 3.31, 95% CI: 2.09, 5.24) and the level of vitamin D in the serum was significantly (*p* = 0.005) lower in patients with PAPS in comparison to healthy individuals (MD: −5.75, 95% CI: −9.73, −1.77) (Figure 3). From the subgroup analysis, interestingly, we observed that the prevalence of thrombotic events in vitamin D deficient PAPS was 62.9% [95% CI: 45.8, 80.0] (Figure S1).

### 3.4. Quality Assessment and Publication Bias

**Figure 4:**
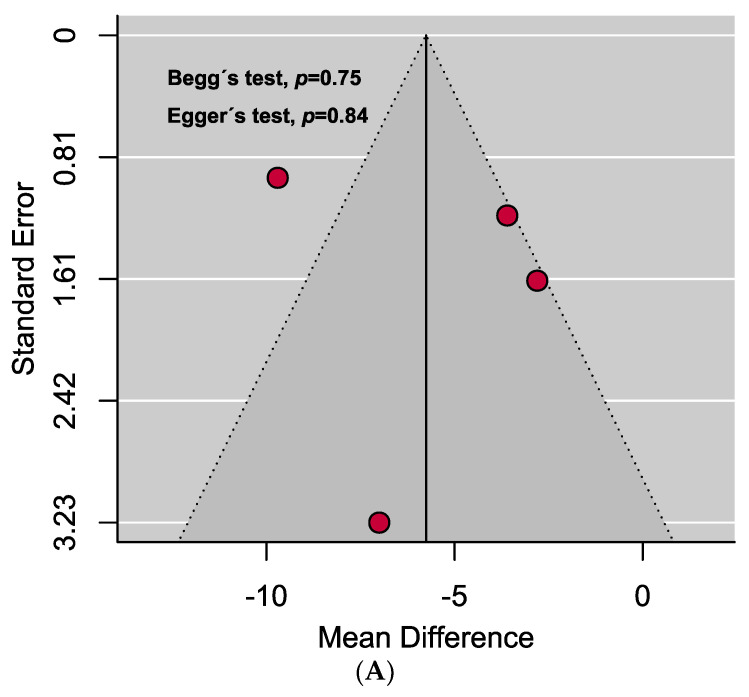

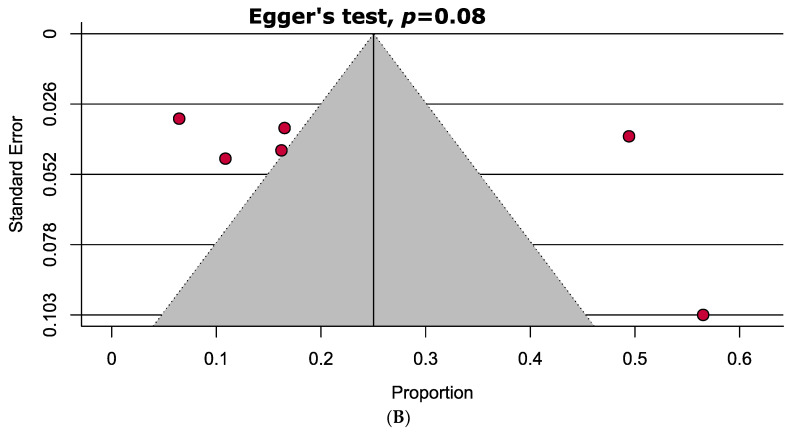
Funnel plots assessing the (**A**) mean difference of serum vitamin D levels in PAPS and controls represents no evidence of publication bias and (**B**) prevalence of vitamin D deficiency in PAPS represents significant publication bias (*p* < 0.10).

A comprehensive overview of the assessment of individual study quality is presented in Tables S4 and S5. In brief, 89% of the eligible studies were high quality (low risk of bias), whereas the methodological quality of only one study was low in this SRMA. Visual inspection of the funnel plot symmetry and quantitative analyses (i.e., Egger’s and Begg’s tests) indicated the absence of publication bias in assessing the mean difference of vitamin D levels in the serum between PAPS and controls. However, significant publication bias was observed in the prevalence of vitamin D deficiency in PAPS (Figure 4).

### 3.5. Sensitivity Analyses

From the sensitivity analyses, it is evident that excluding small studies (<100), excluding low- or medium-quality studies, changing the model into the fixed-effects model or following the leave-one-out method, there were no significant changes in the outcomes when estimating the prevalence (0.7% higher to 10.3% lower) and odds ratio (0.23 to 0.28 lower) of vitamin D deficiency or mean difference of vitamin D levels (0.28 ng/mL lower to 0.84 ng/mL higher) (Table 2, Figure S2). These results indicate that the overall outcomes estimated in this meta-analysis were credible and robust.

## 4. Discussion

Numerous studies have reported on the potential involvement of vitamin D in the pathophysiology of APS [17,19,29,34]. Our findings indicate that there is a higher occurrence of vitamin D insufficiency and deficiency in PAPS. In addition, the likelihood of experiencing vitamin D deficiency is also greater in PAPS when compared to healthy individuals. Our analysis based on the MD also demonstrates a significant association between PAPS and lower serum vitamin D levels in comparison to the control group. This finding corroborates the findings of the previous study [30]. Moreover, the prevalence of thrombotic events in vitamin D deficient PAPS was considerably higher (62.9%, 95% CI: 45.8, 80.0). Interestingly, recent meta-analyses have documented vitamin D deficiency or insufficiency in many of the autoimmune diseases including rheumatoid arthritis [35], ankylosing spondylitis [36], systemic lupus erythematosus (SLE) [37], type 1 diabetes mellitus [38], thyroid disease [39], multiple sclerosis [40], systemic sclerosis [41], and inflammatory bowel disease [42]. Although, in 2018, a meta-analysis and retrospective study on the vitamin D deficiency of APS patients was conducted [30]. Our meta-analysis incorporated comparatively a larger number of studies (*n* = 8) with 1195 participants, while the previous one included only four studies in their quantitative synthesis. In our meta-analysis, we observed a prevalence of vitamin D deficiency in PAPS of 32.2%, which is approximately twice as high as the previous meta-analysis rate of 16.2%. Moreover, we have estimated the prevalence of vitamin D insufficiency in PAPS which was absent from the previous findings. Also, the prevalence of thrombosis (62.9%) in PAPS was found to be higher in our meta-analysis than the previous study (38.9%). Thus, our meta-analysis provides more comprehensive and statistically robust findings on vitamin D status in patients with PAPS. 

Among our included studies, most of the study participants were residents of higher latitude (i.e., European countries, >35° N), which may partly explain the low levels of serum vitamin D in PAPS. There is a documented correlation between increasing latitude and a greater occurrence of vitamin D shortage or insufficiency. This can primarily be attributed to the reduced availability of UVB radiation, which is essential for stimulating the synthesis of vitamin D. This phenomenon is observed not only in the general population residing at higher latitudes but also in individuals with autoimmune disorders [15,43]. As generally, unlike SLE, PAPS patients are not treated with vitamin-D-level-affecting regimens including corticosteroids, hydroxychloroquine or immunosuppressants; the high prevalence and high risk of recurrence of vitamin D deficiency in PAPS are not likely affected by the drugs. Moreover, dietary habit and race were not possibly confounding factors as most of the patients were from European countries who usually intake vitamin-D-rich foods or from Brazil where adequate levels of sun exposure occur throughout the year. Even season might not be the confounding factor as vitamin D deficiency was observed in APS patients during the summer time [29]. As we included only PAPS subjects, and generally, unlike SLE, avoidance of the sun is not prescribed; therefore, avoidance of the sun or the use of sunscreen possibly were not the contributing factors to low vitamin levels in PAPS either. In addition, genetic variation or polymorphism may play a role in causing vitamin D deficiency or insufficiency. Various studies have shown that genetic variations among individuals and populations, such as genetic polymorphisms, might impact the prevalence of vitamin D deficits observed globally [44,45,46,47]. Several single nucleotide polymorphisms (SNPs) in the group-specific component (GC), 25-hydroxylase (CYP2R1), 1-alpha-hydroxylase (CYP27B1), 7-dehydrocholesterol reductase (DHCR7), 24-hydroxylase (CYP24A1), vitamin D binding protein (DBP), and vitamin D receptor (VDR) genes are linked to decreased vitamin D serum levels [44,45,46,47]. Since our study involved participants from Brazil, Italy, Spain, Belarus, and Serbia, we tried to investigate whether any existing studies have reported genetic variants related to vitamin D insufficiency in the populations of these countries. One study discovered an association between the GG genotype of the CYP2R1 (rs10741657) SNP and decreased calcidiol levels in rheumatoid arthritis patients from Spain [48]. Another study measured the genetic risk score (GRS) of Brazilian young adults using six vitamin-D-related SNPs in VDR, DHCR7, CYP2R1, CYP24A1, DBP, and GC, where they found that high vitamin D genetic risk scores were significantly associated with low 25-hydroxyvitamin D concentrations [49]. The findings indicate that genetic factors may influence vitamin D levels, with specific genotypes associated with decreased calcidiol levels. It would be interesting to investigate whether certain genetic variations or polymorphisms play a role in the development of vitamin D insufficiency in individuals with PAPS. Altogether, considering these factors, our findings made us speculate that vitamin D deficiency or insufficiency was probably a part of the complex immune mechanisms that contribute to autoimmune reactions in patients with PAPS [50,51,52].

There is a growing body of evidence that vitamin D may have anticoagulant properties [53,54,55]. This suggests that vitamin D deficiency may contribute to the development of thrombosis. Vitamin D has been demonstrated to downregulate tissue factor, vascular cell adhesion molecule-1, plasminogen activator inhibitor-1, and to upregulate thrombomodulin [56,57,58], indicating the contribution of vitamin D in regulating haemostasis and thrombosis [59]. In addition, vitamin D was considerably lower in thrombotic PAPS (median: 21.7 ng/mL) than obstetric PAPS (33.3 ng/mL) patients [53]. In another retrospective study on PAPS patients, vitamin D insufficiency and deficiency were found in 77.42% and 6.45% of the patients, respectively [31]; however, there was no significant difference between thrombotic and obstetric PAPS manifestations. These results suggest that there may be a relationship between deficiency of vitamin D and the pathogenesis of APS, especially in the thrombotic manifestations in APS.

Although serum levels of vitamin D can vary in different seasons due to UVB rays and low sunlight exposure in the winter as well as a tendency to avoid exposure in the summer [29,60,61,62], there is no data available on seasonal variations in the included studies of this meta-analysis. It was also suggested that certain medications may potentially lead to decreased levels of vitamin D in the serum, in particular; hydroxychloroquine, corticosteroids, or immunosuppressants for autoimmune diseases [63,64]. Nevertheless, there is no data on medications reported in our included studies except one [20], where there was no statistically significant difference in serum vitamin D levels in PAPS patients on a glucocorticoid or without the medication (*p* = 0.93). 

Overall, the factors contributing to heterogeneity in this study may include variations in age, sex, race, geographical locations, seasonal variations, certain, vitamin D supplementation, as well as differences in methods and cut-off values used for measuring vitamin D levels.

## 5. Strengths and Limitations

There are some notable strengths in our systematic review and meta-analysis. As both cross-sectional and case-control studies were included in this meta-analysis, we were able to estimate not only prevalence, but also odds ratio and mean difference (MD) of serum vitamin D levels in patients with PAPS in comparison to healthy individuals. The search strategies were comprehensive and were applied in five of the major databases of medical sciences. The majority of the included studies were high quality (low risk of bias) which indicates the credibility of our findings. There was no publication bias in estimating the MD of vitamin D levels in the serum. Our results were robust and reliable based on the outcomes of the sensitivity analysis. Nevertheless, there were some limitations to address. Only eight studies were included and the number of participants was considerably low. From the included studies, we identified two classification criteria and assay techniques to confirm the patients and measure serum vitamin D levels in PAPS. Moderate to substantial levels of heterogeneity were noted in estimating the prevalence, OR, or MD of vitamin D in PAPS. As the included eight studies encompassed participants from only two continents (i.e., Europe and South America), the results are not representative on a global scale.

## 6. Conclusions

In conclusion, the findings of our meta-analysis indicate that patients with PAPS display considerably lower serum vitamin D levels, higher OR values, higher thrombotic events, and higher vitamin D deficiency or insufficiency in comparison to healthy individuals which is in line with other autoimmune diseases like SLE. These findings suggest that vitamin D supplementation might be beneficial for PAPS patients to maintain stability or even may likely contribute to improve their overall health and potentially reduce disease severity. It is also advisable that clinicians exercise caution while assessing patients with PAPS and consider the possibility of vitamin D deficiency as a mediating factor. Additional investigations are necessary to understand the probable vitamin D deficiency mechanisms and consequences, and ascertain the most effective dosage and duration of vitamin D deficiency treatment in patients with PAPS.

## Figures and Tables

**Table 1:** Major characteristics of the included studies.

Study ID [References]	Country (Latitude)	Study Population	Age of the APS (Mean ± SD/Median [IQR]) (Years)	Disease Duration [Mean ± SD or Median (IQR)] (Months)	APS Classification Criteria	Vitamin D Measurement Method	Cut-Off for Vitamin D Deficiency, Insufficiency (ng/mL)	Summary Outcomes
Riancho-Zarrabeitia 2018 [30]	Spain (40.46° N)	Case: 74 Control: 215	46.1 ± 15.3	NR	2006 Sydney	Chemiluminescent immunoassay	<10, 10–30	Frequency of vitamin D deficiency was found to be higher in APS patients compared to healthy controls. However, no significant difference of vitamin D insufficiency was observed between the healthy control and APS group.
Bećarević 2018 [31]	Serbia (44.81° N)	Case: 62	46.9 ± 12.2	77.9 ± 73.2	2006 Sydney	Chemiluminescent immunoassay	<20, 20–30	Vitamin D levels were significantly lower in female APS patients.
Soroka 2016 [33]	Belarus (53.90° N)	Case: 16	34 ± 6.4	NR	2006 Sydney	ELISA	<15, 20–30	Significant vitamin D deficiency was observed in APS patients.
Andreoli 2012 [29]	Italy (41.87° N)	Case: 115 Control: 128	46.0 [18.0-79.0]	NR	2006 Sydney	Chemiluminescent immunoassay	<10, 10–30	Significantly reduced levels of vitamin D were found in APS patients and thrombotic APS patients showed significantly lower levels of vitamin D than obstetric APS patients.
Agmon-levin 2011 [11]	Italy (41.87° N), Israel (31.04° N), Serbia (44.01° N), Germany (51.16° N), Spain (40.46° N)	Case: 113 Control: 141	48.5 ± 15	NR	2006 Sydney	Chemiluminescent immunoassay	<15, 15–30	In comparison to controls, APS patients had considerably reduced serum vitamin D levels, which were linked to thrombosis.
Paupitz 2010 [20]	Brazil (23.55° S)	Case: 23 Control: 23	33.0 ± 8.2	43.3 ± 35.6	1999 Sapporo	Chemiluminescent immunoassay	<20, 20–30	Lower levels of vitamin D were found in APS patients than controls.
Klack 2010 [32]	Brazil (23.55° S)	Case: 46	40.2 ± 11.9	75.0 ± 56.9	1999 Sapporo	Radioimmunoassay	<10, 10–30	Patients with APS had a very high frequency of vitamin D insufficiency.
Orbach 2007 [17]	Italy (41.87° N), Israel (31.04° N), Hungary (47.16° N)	Case: 160 Control: 141	NR	NR	1999 Sapporo	Radioimmunoassay	<20, 20–30	25-Hydroxy vitamin D levels were relatively lower in APS patients than controls.

APS: antiphospholipid syndrome, IQR: interquartile range; NR: not reported.

**Table 2:** Sensitivity analyses.

Strategies of Sensitivity Analyses	Results	Difference of Pooled Results Compared to the Main Result	Number of Studies Analysed	Total Number of Subjects	Heterogeneity	
					*I^2^*	*p*-Value
**Estimating prevalence of vitamin D deficiency**						
Excluding small studies (<100)	32.9 [0.7, 65.2]	2.2% higher	2	291	98%	<0.01
Excluding low- and medium-quality studies	24.9 [10.0, 39.7]	22.7% lower	6	496	95%	<0.01
Using a fixed-effects model	21.9 [18.8, 25.1]	32.0% lower	7	512	96%	<0.01
**Estimating odds ratio of vitamin D deficiency**						
Excluding small studies (<100)	3.08 [1.89, 5.02]	0.23 lower	3	Case: 365 Control: 484	25%	<0.00001
Excluding low- and medium-quality studies	3.31 [2.09, 5.24]	No change	4	Case: 388 Control: 507	21%	<0.00001
Using a fixed-effects model	3.03 [2.10, 4.37]	0.28 lower	4	Case: 388 Control: 507	60%	<0.00001
**Estimating mean difference of vitamin D level**						
Excluding small studies (<100)	−5.47 [−10.13, −0.82]	0.28 lower	3	Case: 347 Control: 497	91%	<0.00001
Excluding low- and medium-quality studies	−3.62 [−5.43, −1.81]	2.13 lower	3	Case: 210 Control: 379	0%	<0.00001
Using a fixed-effects model	−6.59 [−7.88, −5.30]	0.84 higher	4	Case: 370 Control: 520	87%	<0.00001

## Data Availability

The data presented in this study are available within the article and Supplementary Materials.

## Supplementary Materials

The following supporting information can be downloaded at: https://www.mdpi.com/article/10.3390/antib13010022/s1, Figure S1: Thrombotic prevalence; Figure S2: Sensitivity analyses; Table S1: MOOSE checklist; Table S2: PRISMA checklist; Table S3: Search strategies; Table S4: Quality assessment of Case-control studies; Table S5: Quality assessment of Cross-sectional studies.
