# Supplementary material for: Vitamin D Status in Patients with Primary Antiphospholipid Syndrome (PAPS): A Systematic Review and Meta-Analysis
Source: Antibodies (Basel). 2024 Mar 13;13(1):22. doi: 10.3390/antib13010022 (PMC10967307; doi:10.3390/antib13010022)
Supplement: Supplementary file 1 [file antibodies-13-00022-s001.zip › Figure S2_Sensitivity analyses.pdf]

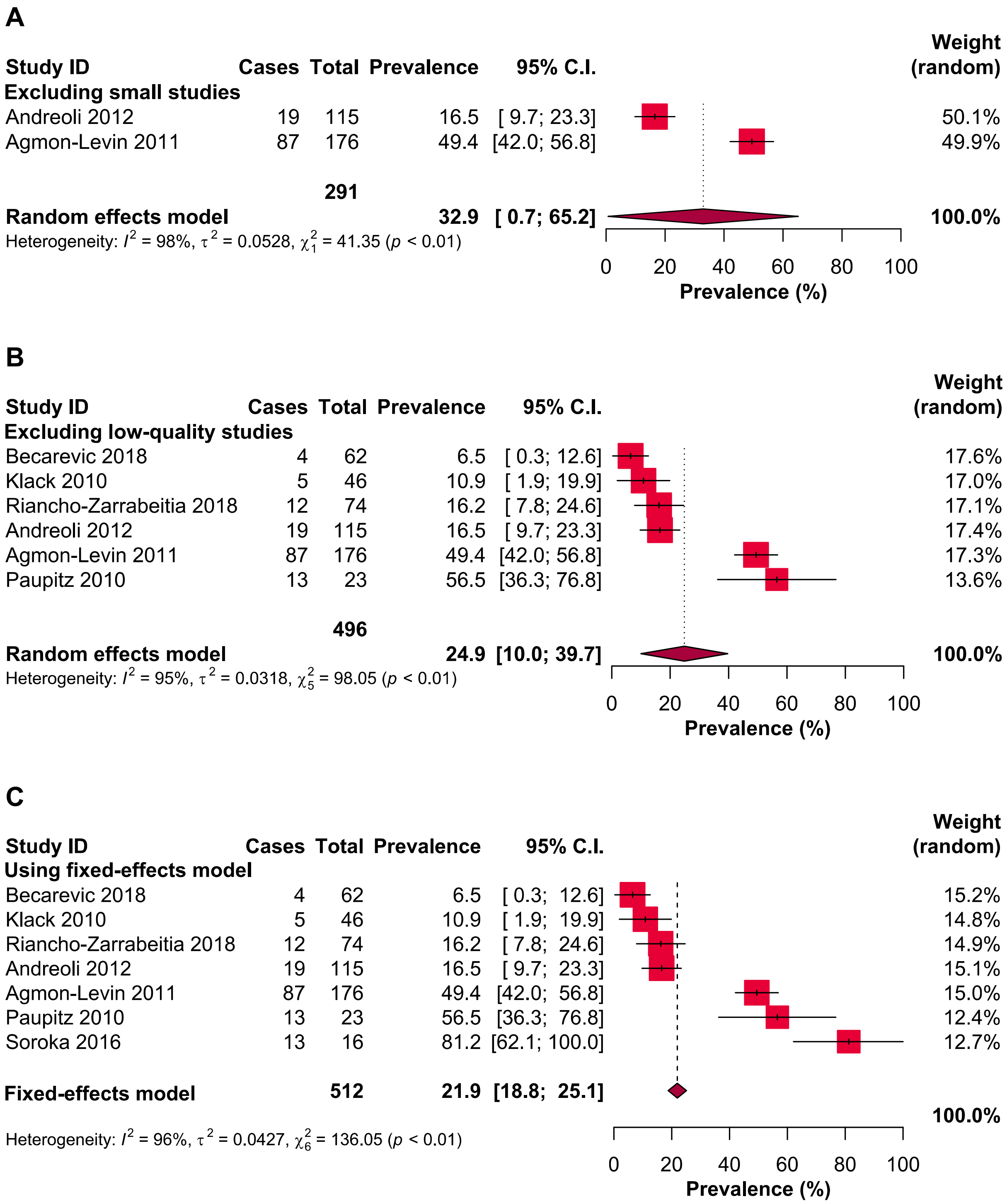
A
Study ID Cases Total Prevalence 95% C.I.
Weight (random)
Excluding small studies
Andreoli 2012 19 115 16.5 [9.7; 23.3] 50.1%
Agmon-Levin 2011 87 176 49.4 [42.0; 56.8] 49.9%
291
Random effects model 32.9 [0.7; 65.2] 100.0%
Heterogeneity: I^2 = 98%, τ^2 = 0.0528, χ^2_1 = 41.35 (p < 0.01)
0 20 40 60 80 100
Prevalence (%)
B
Study ID Cases Total Prevalence 95% C.I.
Weight (random)
Excluding low-quality studies
Becarevic 2018 4 62 6.5 [0.3; 12.6] 17.6%
Klack 2010 5 46 10.9 [1.9; 19.9] 17.0%
Riancho-Zarrabeitia 2018 12 74 16.2 [7.8; 24.6] 17.1%
Andreoli 2012 19 115 16.5 [9.7; 23.3] 17.4%
Agmon-Levin 2011 87 176 49.4 [42.0; 56.8] 17.3%
Paupitz 2010 13 23 56.5 [36.3; 76.8] 13.6%
496
Random effects model 24.9 [10.0; 39.7] 100.0%
Heterogeneity: I^2 = 95%, τ^2 = 0.0318, χ^2_5 = 98.05 (p < 0.01)
0 20 40 60 80 100
Prevalence (%)
C
Study ID Cases Total Prevalence 95% C.I.
Weight (random)
Using fixed-effects model
Becarevic 2018 4 62 6.5 [0.3; 12.6] 15.2%
Klack 2010 5 46 10.9 [1.9; 19.9] 14.8%
Riancho-Zarrabeitia 2018 12 74 16.2 [7.8; 24.6] 14.9%
Andreoli 2012 19 115 16.5 [9.7; 23.3] 15.1%
Agmon-Levin 2011 87 176 49.4 [42.0; 56.8] 15.0%
Paupitz 2010 13 23 56.5 [36.3; 76.8] 12.4%
Soroka 2016 13 16 81.2 [62.1; 100.0] 12.7%
Fixed-effects model 512 21.9 [18.8; 25.1]
100.0%
Heterogeneity: I^2 = 96%, τ^2 = 0.0427, χ^2_6 = 136.05 (p < 0.01)
0 20 40 60 80 100
Prevalence (%)

D

Study or Subgroup	PAPS Events	PAPS Total	Control Events	Control Total	Weight	Odds Ratio M-H, Random, 95% CI
Agmon-Levin 2011	87	176	42	141	56.7%	2.30 [1.45, 3.67]
Andreoli 2012	19	115	6	128	21.3%	4.02 [1.55, 10.47]
Riancho-Zarrabeitia 2018	12	74	8	215	22.0%	5.01 [1.96, 12.80]
**Total (95% CI)**		**365**		**484**	**100.0%**	**3.08 [1.89, 5.02]**
Total events	118		56			

Heterogeneity: Tau² = 0.05; Chi² = 2.68, df = 2 (P = 0.26); I² = 25%
Test for overall effect: Z = 4.51 (P < 0.00001)

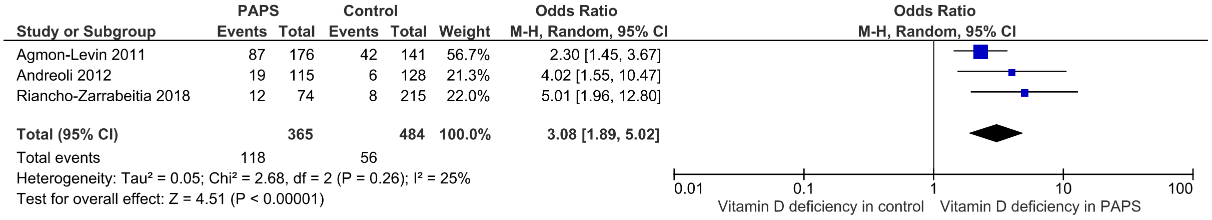

E

Study or Subgroup	PAPS Events	PAPS Total	Control Events	Control Total	Weight	Odds Ratio M-H, Random, 95% CI
Agmon-Levin 2011	87	176	42	141	50.9%	2.30 [1.45, 3.67]
Andreoli 2012	19	115	6	128	19.1%	4.02 [1.55, 10.47]
Paupitz 2010	13	23	4	23	10.4%	6.17 [1.59, 23.99]
Riancho-Zarrabeitia 2018	12	74	8	215	19.6%	5.01 [1.96, 12.80]
**Total (95% CI)**		**388**		**507**	**100.0%**	**3.31 [2.09, 5.24]**
Total events	131		60			

Heterogeneity: Tau² = 0.05; Chi² = 3.82, df = 3 (P = 0.28); I² = 21%
Test for overall effect: Z = 5.09 (P < 0.00001)

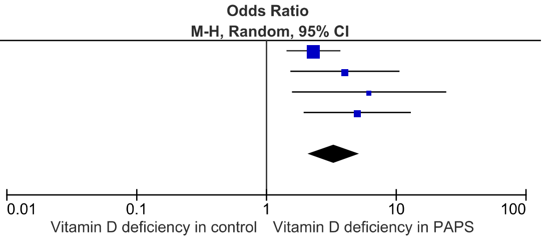

F

Study or Subgroup	PAPS Events	PAPS Total	Control Events	Control Total	Weight	Odds Ratio M-H, Fixed, 95% CI
Agmon-Levin 2011	87	176	42	141	70.4%	2.30 [1.45, 3.67]
Andreoli 2012	19	115	6	128	14.2%	4.02 [1.55, 10.47]
Paupitz 2010	13	23	4	23	5.2%	6.17 [1.59, 23.99]
Riancho-Zarrabeitia 2018	12	74	8	215	10.2%	5.01 [1.96, 12.80]
**Total (95% CI)**		**388**		**507**	**100.0%**	**3.03 [2.10, 4.37]**
Total events	131		60			

Heterogeneity: Chi² = 3.82, df = 3 (P = 0.28); I² = 21%
Test for overall effect: Z = 5.92 (P < 0.00001)

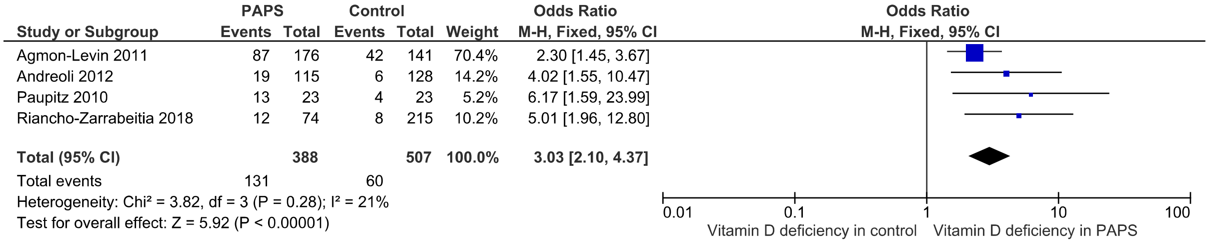

G

Study or Subgroup	PAPS Mean	PAPS SD	PAPS Total	Control Mean	Control SD	Control Total	Weight	Mean Difference IV, Random, 95% CI
Orbach 2007	11.9	5.4	160	21.6	10	141	34.8%	-9.70 [-11.55, -7.85]
Agmon-Levin 2011	18	9	113	21.6	10	141	33.7%	-3.60 [-5.94, -1.26]
Riancho-Zarrabeitia 2018	21.9	11.8	74	24.7	12.8	215	31.4%	-2.80 [-5.99, 0.39]
**Total (95% CI)**			**347**			**497**	**100.0%**	**-5.47 [-10.13, -0.82]**

Heterogeneity: Tau² = 15.32; Chi² = 22.55, df = 2 (P < 0.0001); I² = 91%

Test for overall effect: Z = 2.30 (P = 0.02)

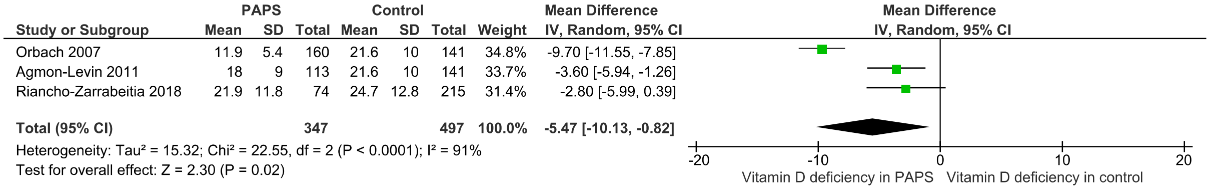

H

Study or Subgroup	PAPS Mean	PAPS SD	PAPS Total	Control Mean	Control SD	Control Total	Weight	Mean Difference IV, Random, 95% CI
Paupitz 2010	21.6	11.2	23	28.6	10.7	23	8.2%	-7.00 [-13.33, -0.67]
Agmon-Levin 2011	18	9	113	21.6	10	141	59.7%	-3.60 [-5.94, -1.26]
Riancho-Zarrabeitia 2018	21.9	11.8	74	24.7	12.8	215	32.2%	-2.80 [-5.99, 0.39]
**Total (95% CI)**			**210**			**379**	**100.0%**	**-3.62 [-5.43, -1.81]**

Heterogeneity: Tau² = 0.00; Chi² = 1.35, df = 2 (P = 0.51); I² = 0%

Test for overall effect: Z = 3.92 (P < 0.0001)

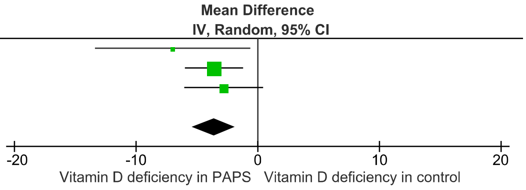

I

Study or Subgroup	PAPS Mean	PAPS SD	PAPS Total	Control Mean	Control SD	Control Total	Weight	Mean Difference IV, Fixed, 95% CI
Orbach 2007	11.9	5.4	160	21.6	10	141	48.8%	-9.70 [-11.55, -7.85]
Paupitz 2010	21.6	11.2	23	28.6	10.7	23	4.2%	-7.00 [-13.33, -0.67]
Agmon-Levin 2011	18	9	113	21.6	10	141	30.5%	-3.60 [-5.94, -1.26]
Riancho-Zarrabeitia 2018	21.9	11.8	74	24.7	12.8	215	16.5%	-2.80 [-5.99, 0.39]
**Total (95% CI)**			**370**			**520**	**100.0%**	**-6.59 [-7.88, -5.30]**

Heterogeneity: Chi² = 22.57, df = 3 (P < 0.0001); I² = 87%

Test for overall effect: Z = 9.99 (P < 0.00001)

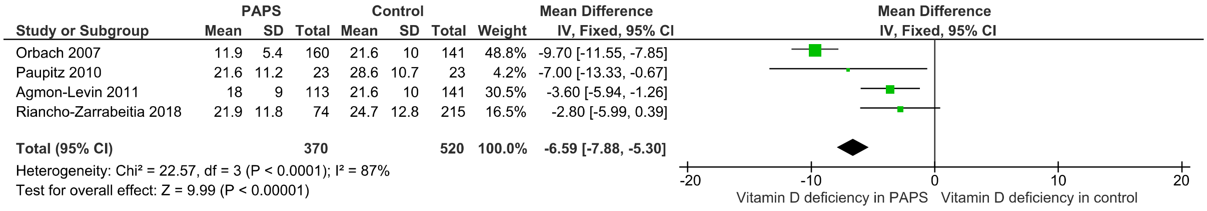

Figure S2. Sensitivity analysis. Estimating prevalence (A) excluding small studies (<100), (B) excluding low- and medium-quality studies, (C) using a fixed-effects model; estimating risk ratio (D) excluding small studies (<100), (E) excluding low- and medium-quality studies, (F) using a fixed-effects model and estimating mean difference (G) excluding small studies (<100), (H) excluding low- and medium-quality studies, and (I) using a fixed-effects model.
