# Supplementary material for: Vitamin D Status in Patients with Primary Antiphospholipid Syndrome (PAPS): A Systematic Review and Meta-Analysis
Source: Antibodies (Basel). 2024 Mar 13;13(1):22. doi: 10.3390/antib13010022 (PMC10967307; doi:10.3390/antib13010022)
Supplement: Supplementary file 1 [file antibodies-13-00022-s001.zip › Table S1_MOOSE checklist.pdf]

Supplementary Materials

Table S1. MOOSE Checklist

Item No	Criteria	Sentences mentioned in the meta-analysis or appropriate explanations
1	Problem definition	High prevalence of low serum levels of vitamin D has been observed worldwide involving both healthy and ill subjects including patients with rheumatic diseases. Vitamin D insufficiency and/or deficiency have been observed in many autoimmune diseases like rheumatoid arthritis, Sjögren syndrome, SLE, Behçet's disease, multiple sclerosis and systemic sclerosi. Additionally, serum levels of vitamin D were observed to be inversely associated with thrombotic events in patients with APS.
2	Hypothesis statement	Although there is a growing body of evidence indicating the tendency of manifesting low serum levels of vitamin D in patients with APS, it is inconclusive.
3	Description of study outcome(s)	To evaluate the serum levels of vitamin D in patients with PAPS in compared to healthy controls.
4	Type of exposure or intervention used	Serum levels of vitamin D.
5	Type of study designs used	Case-control & Cross-sectional studies.
6	Study population	APS of adult age (≥18 years), of any sex or race were considered eligible patients. Adult healthy subjects (≥18 years) of any sex or race were considered eligible control

		participants.
7	Qualifications of searchers (e.g., librarians and investigators)	M.A.I. (PhD), S.A. (MSc), S.S. (MSc), S.S.A. (BSc)
8	Search strategy, including time period included in the synthesis and keywords	Electronic databases including PubMed, Scopus, Web of Science, ScienceDirect and Google Scholar were searched and screened independently by three authors (M.A.I, S.A. and S.S.A.). The final systematic search was conducted on May 31, 2023. There were no year and language restrictions.<br><br>PubMed. ((((antiphospholipid[Title/Abstract]) OR anti-phospholipid[Title/Abstract]) OR APS[Title/Abstract])) AND ((((("vitamin D"[Title/Abstract]) OR "25-hydroxyvitamin D"[Title/Abstract]) OR "25(OH)D"[Title/Abstract]) OR Cholecalciferol[Title/Abstract]) OR Ergocalciferol[Title/Abstract]) OR Calcifediol[Title/Abstract])<br><br>Scopus. TITLE(antiphospholipid OR anti-phospholipid OR APS) AND TITLE("vitamin D" OR "25-hydroxyvitamin D" OR "25(OH)D" OR Cholecalciferol OR Ergocalciferol OR Calcifediol)<br><br>ScienceDirect. Title, abstract, keywords: (antiphospholipid OR anti-phospholipid OR APS) AND ("vitamin D" OR "25-hydroxyvitamin D" OR "25(OH)D" OR Cholecalciferol OR Ergocalciferol OR Calcifediol)

		Web of Science. TI=(antiphospholipid OR anti-phospholipid OR anti-phospolipid OR APS) AND TI=("vitamin D" OR "25 OH D" OR hypovitaminosis OR hydroxyvitamin)<br><br>Google Scholar. allintitle: (antiphospholipid OR anti-phospholipid OR APS) ("vitamin D" OR "25-hydroxyvitamin D" OR "25(OH)D" OR Cholecalciferol OR Ergocalciferol OR Calcifediol)
9	Effort to include all available studies, including contact with authors	Three authors (M.A.I., S.A. and S.S.A.) took part in the discussion to resolve any discrepancies, unclear or missing data presentation. If unresolved, either the corresponding or the first author of the respective study was contacted for further clarifications.
10	Databases and registries searched	Electronic databases including PubMed, Scopus, Web of Science, ScienceDirect and Google Scholar were searched
11	Search software used, name and version, including special features used (*e.g.*, explosion)	Duplicate studies which may result from different electronic databases were removed and managed by EndNote software (version X8).
12	Use of hand searching (*e.g.*, reference lists of obtained articles)	In addition, references in the primary selected studies were also examined to identify any other possible relevant studies.
13	List of citations located and those excluded, including justification	Table 1 and Supplementary Table S3
14	Method of addressing articles	There were no year and language restrictions.

	published in languages other than English	However, no articles were found other than in English.
15	Method of handling abstracts and unpublished studies	We did not consider unpublished studies to be included in the analysis.
16	Description of any contact with authors	Prof. Dr. Yehuda Shoenfeld was contacted about the raw data of the included study Orbach 2007.
17	Description of relevance or appropriateness of studies assembled for assessing the hypothesis to be tested	Detailed inclusion and exclusion criteria were described in "Eligibility criteria" of "Methods" section.
18	Rationale for the selection and coding of data (*e.g.*, sound clinical principles or convenience)	Selection of the studies was based on the inclusion and exclusion criteria.
19	Documentation of how data were classified and coded (*e.g.*, multiple raters, blinding and interrater reliability)	Mentioned in the "Data extraction" section.
20	Assessment of confounding (e.g., comparability of cases and controls in studies where appropriate)	No such analysis could be done.
21	Assessment of study quality, including blinding of quality assessors, stratification or regression on possible predictors of study results	Quality assessment of each of the included studies was evaluated by three authors (MAI, SA and SSA) based on the Joana Brigg's Institute (JBI) protocol.
22	Assessment of heterogeneity	To assess the heterogeneity (I^2) of the included studies, Chi-squared test was used where I^2 assessed the quantity of inconsistency across the studies ($p<0.10$ was considered as significant). A value of I^2 close to zero indicates homogeneity, whereas, the

		following ranges of I² were used to interpret heterogeneity: low heterogeneity if I²=25-50%, moderate heterogeneity if I²=51-75% and substantial heterogeneity if I²>75%.
23	Description of statistical methods (*e.g.*, complete description of fixed or random effects models, justification of whether the chosen models account for predictors of study results, dose-response models, or cumulative meta-analysis) in sufficient detail to be replicated	Described in the “Methods” section.
24	Provision of appropriate tables and graphics	We included PRISMA flow diagram (Fig. 1), main forest plots (Fig. 2), Trim and fill funnel plot (Fig. 4), forest plots for sensitivity analyses (Fig. S2), major characteristics table (Table 1), sensitivity analyses table (Table 2) and table of excluded studies (Supplementary Table S3).
25	Graphic summarizing individual study estimates and overall estimate	Main forest plots (Fig. 2).
26	Table giving descriptive information for each study included	Major characteristics table (Table 1).
27	Results of sensitivity testing (*e.g.*, subgroup analysis)	Fig. S2 & Table 2.
28	Indication of statistical uncertainty of findings	95% confidence intervals were presented with all summary estimates, I^2 values and results of subgroup and sensitivity analyses.
29	Quantitative assessment of bias (*e.g.*, publication bias)	Funnel plot (Fig. 4).
30	Justification for exclusion (*e.g.*,	Supplementary Table S3. Studies excluded

	exclusion of non-English language citations)	from the meta-analysis.
31	Assessment of quality of included studies	Risk of bias assessment table (Table S4 & S5).
32	Consideration of alternative explanations for observed results	Discussed in the “Discussion” section.
33	Generalization of the conclusions (i.e., appropriate for the data presented and within the domain of the literature review)	Discussed in the “Discussion” section.
34	Guidelines for future research	Discussed in the “Discussion” section.
35	Disclosure of funding source	None to declare.
