# Supplementary material for: Vitamin D Status in Patients with Primary Antiphospholipid Syndrome (PAPS): A Systematic Review and Meta-Analysis
Source: Antibodies (Basel). 2024 Mar 13;13(1):22. doi: 10.3390/antib13010022 (PMC10967307; doi:10.3390/antib13010022)
Supplement: Supplementary file 1 [file antibodies-13-00022-s001.zip › Table S3_Search strategies.pdf]

Table S3. Search strategies

Databases	Search strategies
PubMed	(antiphospholipid[Title/Abstract] OR anti-phospholipid[Title/Abstract] OR anti-phospolipid[Title/Abstract] OR APS[Title/Abstract]) AND ("vitamin D"[Title/Abstract] OR "25 OH D"[Title/Abstract] OR hypovitaminosis[Title/Abstract] OR hydroxyvitamin[Title/Abstract])
Web of Science	TI=(antiphospholipid OR anti-phospholipid OR anti-phospolipid OR APS) AND TI=("vitamin D" OR "25 OH D" OR hypovitaminosis OR hydroxyvitamin)
Scopus	TITLE(antiphospholipid OR anti-phospholipid OR anti-phospolipid OR APS) AND TITLE-ABS("vitamin D" OR "25 OH D" OR hypovitaminosis OR hydroxyvitamin)
Google Scholar	allintitle:(antiphospholipid OR anti-phospholipid OR anti-phospolipid OR APS) ("vitamin D" OR "25 OH D" OR hypovitaminosis OR hydroxyvitamin)
ScienceDirect	Title, abstract, keywords: (antiphospholipid OR anti-phospholipid OR anti-phospolipid OR APS) AND ("vitamin D" OR "25 OH D" OR hypovitaminosis OR hydroxyvitamin)
