# Supplementary material for: Vitamin D Status in Patients with Primary Antiphospholipid Syndrome (PAPS): A Systematic Review and Meta-Analysis
Source: Antibodies (Basel). 2024 Mar 13;13(1):22. doi: 10.3390/antib13010022 (PMC10967307; doi:10.3390/antib13010022)
Supplement: Supplementary file 1 [file antibodies-13-00022-s001.zip › Table S4_Quality assessment of Case-control studies.pdf]

Table S4. Quality assessment of the included case-control studies

Study ID	1	2	3	4	5	6	7	8	9	10	Yes
Agmon-levin 2011	Y	Y	Y	Y	Y	U	U	Y	Y	Y	80%
Andreoli 2012	Y	Y	Y	Y	Y	Y	U	Y	Y	Y	90%
Orbach 2007	Y	U	U	Y	Y	U	U	Y	U	Y	50%
Paupitz 2010	Y	Y	Y	Y	Y	U	U	Y	Y	Y	80%
Riancho-Zarrabeitia 2018	Y	Y	Y	Y	Y	U	U	Y	Y	Y	80%

1. Were the groups comparable other than the presence of disease in cases or the absence of disease in controls? 2. Were cases and controls matched appropriately? 3. Were the same criteria used for identification of cases and controls? 4. Was exposure measured in a standard, valid and reliable way? 5. Was exposure measured in the same way for cases and controls? 6. Were confounding factors identified? 7. Were strategies to deal with confounding factors stated? 8. Were outcomes assessed in a standard, valid and reliable way for cases and controls? 9. Was the exposure period of interest long enough to be meaningful? 10. Was appropriate statistical analysis used? Y: Yes, N: No, U: Unclear, NA: Not applicable
