# Supplementary material for: Vitamin D Status in Patients with Primary Antiphospholipid Syndrome (PAPS): A Systematic Review and Meta-Analysis
Source: Antibodies (Basel). 2024 Mar 13;13(1):22. doi: 10.3390/antib13010022 (PMC10967307; doi:10.3390/antib13010022)
Supplement: Supplementary file 1 [file antibodies-13-00022-s001.zip › Table S5_Quality assessment of Cross-sectional studies.pdf]

Table S5. Quality assessment of the included cross-sectional studies

Study ID	1	2	3	4	5	6	7	8	Yes
Bećarević 2018	Y	Y	Y	Y	U	U	Y	Y	75%
Klack 2010	Y	Y	Y	Y	U	U	Y	Y	75%
Soroka 2016	Y	Y	Y	Y	U	U	Y	Y	75%

1. Were the criteria for inclusion in the sample clearly defined? 2. Were the study subjects and the setting described in detail? 3. Was the exposure measured in a valid and reliable way? 4. Were objective, standard criteria used for measurement of the condition? 5. Were confounding factors identified? 6. Were strategies to deal with confounding factors stated? 7. Were the outcomes measured in a valid and reliable way? 8. Was appropriate statistical analysis used? Y: Yes, N: No, U: Unclear, NA: Not applicable
